# Identifying a science‐based methodology for generating refined maps that identify where pesticides overlap with endangered/threatened species habitat

**DOI:** 10.1002/jeq2.70134

**Published:** 2026-01-09

**Authors:** Taylor Randell‐Singleton, A. Stanley Culpepper

**Affiliations:** ^1^ Department of Crop and Soil Sciences University of Georgia Tifton Georgia USA

## Abstract

To manage pests, including problematic weeds, farmers rely on an integrated pest management approach that encourages the safe and effective use of pesticides. As US federal regulators place restrictions on pesticides as a means of protecting endangered and threatened (known collectively as listed) species, it is paramount these decisions are based on empirical evidence. During 2022, restrictions placed on the Enlist Duo (glyphosate + 2,4‐D choline) herbicide to protect listed species lacked scientific merit. As a means of protecting the reticulated or frosted flatwoods salamander, the ability to use the herbicide was lost to many farmers across the country, including those in 11 Georgia counties. County‐wide restrictions in Georgia, based on the historical presence of these salamanders, prevented its use on over 366,000 ha (904,000 acres) of corn, cotton, and soybean. Using spatial datasets to identify specific salamander habitat and where it overlapped with potential pesticide applications, results documented that less than 1% of impacted hectares should even be included in the restrictions; over 99% of the restricted farmland did not provide suitable habitat features needed for the species’ survival. This research confirms the importance of utilizing a science‐based mapping methodology for future pesticide restrictions being implemented to protect listed species.

## Core Ideas

- Pesticide use restrictions lacking scientific merit limit a farmer's ability to protect crops and meet world food supply demands.
- Restrictions are being placed on pesticides to protect listed species; it is paramount they are a result of unbiased sound science.
- Understanding where pesticide applications and sensitive species may intersect is critical to protecting both the farm and wildlife.
- We propose methodology that bases pesticide restrictions on locations of suitable habitat rather than on historical presence.
- This methodology can be implemented as a nationwide approach, fostering species protection and maintaining practical pesticide use.

## Plain Language Summary

The use of pesticides to protect crops from pests is a critical part of ensuring agricultural sustainability and stable food supplies worldwide. As federal regulations are placed on pesticides as a means of protecting endangered and threatened species, it is critical that these decisions are science‐based. Restrictions placed on Enlist Duo (2,4‐D choline + glyphosate) during 2022 eliminated use on corn, cotton, and soybeans in 11 Georgia counties, impacting over 366,000 ha. To better understand the impact of these restrictions, which were based on the historical presence of two sensitive species (frosted and reticulated flatwoods salamanders), spatial datasets were utilized to create maps that identified specific habitat parameters and their interactions with pesticide use sites. Results indicate that a science‐based mapping methodology can limit pesticide use restrictions to exactly where listed species are located, conserving species and habitat, while enabling production agriculture to continue meeting the demands of increasing populations.

**Abbreviations**
CDL: cropland data layer
EPA: Environmental Protection Agency
ESA: Endangered Species Act
FIFRA: Federal Insecticide, Fungicide, and Rodenticide Act
FWS: Fish and Wildlife Service
NBD: National Boundary Dataset
NMFS: National Marine Fisheries Service
USDA: United States Department of Agriculture
USGS: United States Geological Survey

## 1 INTRODUCTION

Agricultural sustainability, or the ability to provide the food, feed, and fiber that supports the basic needs of a rapidly growing world population, is built upon a foundation of pesticide use to sustain and maximize production (Cooper & Dobson, 2007; Washuck et al., 2022). Problematic pests, including weeds, insects, and diseases, are constantly competing with crops for valuable resources, threatening the ability to produce a bountiful and economical yield (US Department of Agriculture [USDA], 2023). The ability to protect vulnerable crops from these risks is a critical step in ensuring stability of food supplies worldwide (CropLife International, 2025). While the use of pesticides is often a controversial topic, significant research has highlighted both the human and environmental safety of proper pesticide use in an integrated pest management program, along with the necessity of utilizing these tools to produce an economically and environmentally sustainable food supply (Cooper & Dobson, 2007; Reeves et al., 2019). Recently, attention in the United States has focused on better understanding interactions between pesticide use and the surrounding environment, particularly on their impacts on nontarget species such as endangered and threatened species (US Environmental Protection Agency [US EPA], 2022a, 2022b).

When the Endangered Species Act (ESA) was enacted as a federal law in the United States during 1973, its primary objectives were to (1) ensure protection for vulnerable species at risk of extinction, (2) provide the conservation framework needed to protect the species from harm, and (3) offer the opportunity to recover to the point that population numbers were once again stable (Bean, 2009; Schwartz, 2008). By listing species and their critical habitat as endangered or threatened (together they are known collectively as listed species), this offers protections from actions conducted by federal agencies that may “jeopardize the continued existence of these species or result in the destruction or adverse modification of designated habitat” (Bean, 2009; Easter‐Pilcher, 1996; Schwartz, 2008). Considering the intersection of production agriculture and the ESA, federal actions that have the potential to impact listed species include registration, reregistration, and regulation of pesticide use by the Environmental Protection Agency (EPA) under the Federal Insecticide, Fungicide, and Rodenticide Act (FIFRA). Under the statute of the law, each FIFRA action must also comply with the ESA, ensuring that all registered uses of pesticides within each cropping system do not adversely impact any listed species or their habitat, including any associated feeding sources, breeding, and shelter sites (US Environmental Protection Agency [US EPA], 2022a, 2022b).

With over 1600 listed species, 900 critical habitats, and over 1200 pesticide active ingredients registered to date, the time‐ and resource‐intensive analysis between pesticides and listed species has unfortunately been overlooked for decades. This has led to the need for a better understanding of potential interactions between the locations of listed species and pesticide use sites across the country (US Fish and Wildlife Service [US FWS], 2025). Furthermore, when the EPA registers a pesticide that could potentially harm a listed species, a consultation with the US Fish and Wildlife Services (FWSs) and the National Marine Fisheries Services (NMFSs) is required. Routinely, this consultation process between FWS and NMFS, the two federal agencies that oversee listed species protection, has been inefficient at best and unsuccessful at worst. The result of these inactions has left listed species potentially susceptible to negative effects, along with increasing legal vulnerability of the pesticides that farmers rely on to ensure they can produce a viable crop (US Environmental Protection Agency [US EPA], 2025). Lawsuits have challenged this lack of compliance, which increases uncertainties surrounding pesticide availability for growers around the country. A recently published paper by The Council for Agricultural Science and Technology (CAST) (2024) has explored this issue in greater depth.

In an effort to protect listed species under ESA while minimizing the risk of litigation, the label for Enlist Duo released during 2022 prohibited the use of this product for many farmers across the United States (Corteva Agriscience, 2022). This herbicide contains a mixture of the active ingredients 2,4‐D choline and glyphosate and was historically used across the country in tolerant corn, cotton, and soybean varieties for the control of emerged weeds. The basis of these restrictions was an effort to proactively prevent exposure of listed species to potential Enlist Duo spray drift and runoff. Specifically for Georgia, the label prevented its use across 11 Georgia counties in their entirety.

The rationale behind the preemptive Enlist Duo restrictions in Georgia was for the protection of two endangered species considered to be particularly sensitive to the product, the frosted flatwoods salamander (*Ambystoma cingulatum*) and the reticulated flatwoods salamander (*Ambystoma bishopi*). Native to the Southern Coastal Plain of the United States, these species reside within a highly specific habitat of the longleaf pine wiregrass ecosystem found in Southern Georgia. Historically, one or both of these species have been recorded in each of the 11 impacted counties. Regardless of the age of the record for the most recent observation of the species in the county or the evaluation of current suitability of these sites to sustain the endangered salamanders, restrictions were placed on the label without an in‐depth analysis of the potential interactions between the pesticide use and locations of the species. With over a combined 721,086 ha (1781,842 acres) of corn, cotton, and soybean across the entire state of Georgia annually (University of Georgia [UGA], 2024), pesticide use restrictions based on geopolitical boundaries (such as county or state boundaries) that do not consider the specific features and characteristics that constitute suitable habitat of a listed species potentially place overly conservative limitations on farmers and land managers; this threatens their ability to manage pests and remain economically sustainable (University of Georgia [UGA], 2025). Equally important, it is imperative that our most sensitive and vulnerable species and habitats are protected from threats and negative pressures outside their native ecosystems. Accomplishing these tasks begins with understanding where the species and their habitats are located (Dunne et al., 2023, 2025)

Preliminary information collected by University of Georgia Extension suggests that county‐wide pesticide use restrictions, which were imposed by the 2022 Enlist Duo label, were unnecessarily prohibitive for protecting listed species, considering that most species are not located near agriculture fields in the state and aggressive off‐field pesticide movement mitigation practices are already in place (Culpepper et al., 2020). To support this premise, a team consisting of representatives from the University of Georgia Cooperative Extension, the Georgia Department of Agriculture, and Georgia farmers was formed to address the need for more refined information that identified where listed species are actually located in relation to where pesticides are currently applied to farmland around the state.

Working in cooperation with the US EPA and regional US FWS representatives (including flatwoods salamander species experts), the team's goal was to develop a scientific methodology for identifying potential locations of the two salamander species and their associated suitable habitat in relation to agricultural pesticide use sites. The methodology would offer the opportunity to increase knowledge and awareness about these sensitive species around the state, while also ensuring that practical pesticide use could continue where appropriate. This dual‐part goal, of both protecting listed species and ensuring growers could continue using the pesticide products essential to producing a viable crop, offered a tremendous and unique opportunity for all involved entities to learn more about ongoing efforts and challenges facing the various disciplines represented within this cooperative team, therefore informing efforts that concurrently support both sustainable food production and healthy environments.

Following collaborations with regional FWS experts to understand the land features and characteristics that comprise suitable habitat for the frosted flatwoods salamander and reticulated flatwoods salamander (hereon collectively referred to as flatwoods salamanders), and identifying spatial datasets that highlight these features, the objectives of this working group were to (1) calculate the number of hectares and associated economic value of Georgia corn, cotton, and soybean impacted by Enlist Duo restrictions, (2) identify locations of flatwoods salamander species in Georgia as provided by historical location information, (3) work with FWS to develop maps of suitable salamander habitat using parameters defined by species experts, and (4) create a methodology that utilizes spatial data to identify specific sites where Enlist Duo use may potentially overlap with historical and newly defined suitable habitat of the flatwoods salamander species.

**Core Ideas**

- Pesticide use restrictions lacking scientific merit limit a farmer's ability to protect crops and meet world food supply demands.
- Restrictions are being placed on pesticides to protect listed species; it is paramount they are a result of unbiased sound science.
- Understanding where pesticide applications and sensitive species may intersect is critical to protecting both the farm and wildlife.
- We propose methodology that bases pesticide restrictions on locations of suitable habitat rather than on historical presence.
- This methodology can be implemented as a nationwide approach, fostering species protection and maintaining practical pesticide use.

## 2 MATERIALS AND METHODS

To better understand impacted cropland and historical locations of species, build habitat maps, and generate a methodology for identifying overlap between pesticide use and listed species habitat, multiple publicly available spatial datasets were accessed and utilized for analysis. Throughout the project, all spatial analysis, area calculations, and habitat map building were conducted in ArcPro GIS software (Esri, 2024). The respective datasets utilized, file formatting, sources, and data processing steps are outlined in subsequent sections.

### 2.1 Cropland hectares impacted

To calculate the impact this ESA regulation had on Georgia farmers through Enlist Duo label restrictions, both the hectares of cropland no longer able to be treated with the herbicide and the value of those crops were determined. First, the 11 Georgia counties that could no longer use Enlist Duo were identified from the label; those counties included Baker, Berrien, Brooks, Burke, Calhoun, Early, Irwin, Lee, Miller, Screven, and Worth counties (Corteva Agriscience, 2022). Next, the United States Geological Survey (USGS) National Boundary Dataset (NBD) dataset, which represents USGS governmental boundaries for states and counties, was utilized to delineate each county boundary, defining the restricted areas on the project GIS map. The NBD spatial dataset is updated annually; the 2021 published shapefile was utilized for the purposes of this project (US Geological Survey [USGS], 2022).

Once the county boundaries were defined, the field locations of corn, cotton, and soybean crop fields were identified within those 11 counties through the United States Department of Agriculture (USDA) National Agricultural Statistics Service Cropland Data Layer (CDL) spatial dataset. The CDL is a robust raster‐based dataset updated annually, which utilizes satellite imagery to identify and record the geographic field location (presented as pixels) of over 100 different categories of crops across the country. To capture field location and associated hectares associated with crop rotations over time, the CDL was assessed over a cumulative 5‐year period from 2017 through 2021, where data were exported as raster files for each county/year combination for further processing. Double crop scenarios containing the specified crops were included when available; years and descriptions of double crop scenarios are included in Table 1.

**TABLE 1:** United States Department of Agriculture (USDA) cropland data layer (CDL) year availability for data of field locations from double crop scenarios containing corn, cotton, and soybean. Data from available years were downloaded and processed for map incorporation.

Fiedl location	WinWht/soybeans	WinWht/corn	Oats/corn	WinWht/cotton	Soybeans/oats	Corn/soybeans	Barley/soybeans
Baker	2017–2021	2017–2019; 2021	2017–2018; 2020–2021	2017–2021	2017–2018; 2021	2017–2018; 2020	–
Berrien	2017–2021	2019–2021	2017–2018; 2020–2021	2017–2021	2017–2021	2017–2021	–
Brooks	2017–2021	2017; 2019–2021	2017–2019; 2021	2017–2021	2017–2021	2017–2021	2020
Burke	2017–2018; 2021	2017–2021	2017–2021	2017–2021	2017–2021	2018–2019; 2021	2017; 2020–2021
Calhoun	2017–2021	2017–2021	2018–2021	2017–2021	2017–2021	2017–2018	2021
Early	2017–2021	2017–2021	2017–2021	2017–2021	2017–2021	2017–2018	–
Irwin	2017–2021	2017; 2019–2021	2017–2018; 2020–2021	2017–2021	2017–2021	2017–2018	2021
Lee	2017–2021	2017; 2019–2021	2017–2018; 2020–2021	2017–2021	2017–2021	2017–2018	2021
Miller	2017–2021	2017–2021	2018; 2020–2021	2017–2021	2017–2021	2017–2018	–
Screven	2017–2021	2020–2021	2019–2021	2017–2021	2017–2021	2019; 2021	2020
Worth	2017–2021	2017; 2019–2021	2017–2018; 2020–2021	2017–2021	2017–2021	2017–2019; 2021	–

Abbreviation: WinWht, winter wheat.

For analysis, each individual raster file was first converted to a polygon file type. If attributes were not already attached to the file, then metadata were downloaded from the USDA Cropland CROS webpage (US Department of Agriculture [USDA], 2022) and joined to the polygon. The newly created polygons were split by attribute of class name (representing crop type), which created a new polygon file for each type of crop present within the constraints of the original file. Each file was given a similar naming structure to maintain file integrity, which included both the county name, year, and crop type (ex. baker_2017_cotton).

Due to the nature in which the national CDL dataset is generated using satellite imagery, rogue pixels presenting as crop fields may incorrectly appear on the landscape imagery, representing a false potential pesticide use site or crop field. Based on previously developed methodology by the authors, for the purposes of this project any CDL pixel less than 0.81 ha (2 acres) in size was removed from the dataset (Randell‐Singleton & Culpepper, 2024).

Once CDL raster files were processed, all crops of interest for each county during the five cumulative evaluated years were added back to the map. Each of these layers was merged by county to create a single cohesive layer of all combined croplands over the 5‐year period in the respective county, and file boundaries were dissolved to create united polygon files. Within the attribute table of each polygon file, hectares were calculated using spatial geometry measurement tools in the GIS software, which enabled data summarization.

The economic value of corn, cotton, and soybean was determined based on reported hectares and associated summarized dollar value for each impacted crop and county in Georgia, averaged over the 5‐year CDL range. For each year (2017–2021) and county, the economic value associated with the respective crop was accessed from the University of Georgia Farm Gate Value Report, which represents summarized data on cropping systems and subsequent total value from agricultural entities across the state (University of Georgia [UGA], 2018, 2019, 2020, 2021, 2022).

### 2.2 Historical population location determination

While the precise location of federally listed species is protected, record of imprecise locations of historical populations within the state of Georgia is accessible through the Georgia Department of Natural Resources Biodiversity Data Portal (Georgia Department of Natural Resources [GA DNR], 2025). This database contains generalized locations of listed species in Georgia and are provided on a fuzzed quarter quad scale (1/4 of a USGS 7.5‐min quadrangle) to protect the exact location of the record. As new information becomes available through surveys, historical records, or observations, the database is updated; for purposes of the project, the locations of each salamander species were accessed from the most recently available dataset at the time of the project (May 2023), which remains relevant to date. A shapefile for each species was downloaded from the biodiversity portal and imported into GIS, maintaining the integrity of the location information and associated attributes.

Shapefiles providing the fuzzed historical locations and record of both flatwoods salamander species in Georgia were imported to the same map containing county boundaries and processed cropland files. Each species shapefile was clipped to the boundaries of the impacted counties, and overlap determinations were made between historical population sites and any cropland that intersected these areas. The spatial intersect tool in GIS was utilized for overlap determinations and geometry calculation processing was utilized to calculate hectares for data summarization within the attribute table of each polygon file.

### 2.3 Development of new salamander habitat maps

Working in cooperation with regional US FWS species experts, detailed vegetation and land cover pattern preferences for the two flatwoods salamander species were defined, and publicly available spatial datasets were identified that would allow for classification of these habitat parameters on the landscape. Land cover data used in the map creation were downloaded from the USGS GAP/LANDFIRE National Terrestrial Ecosystems dataset, which includes detailed vegetation and land cover patterns for the continental United States. Land cover information for Georgia was specifically downloaded from the most recently available dataset at the time of the project (2011).

The GAP/LANDFIRE dataset includes 584 unique land classifications, which is the most refined level of detail available within the dataset; therefore, the NatureServe database (NatureServe, 2025) was cross‐referenced to determine which land classifications (represented and titled as ecological systems) were identified as suitable habitat for the species. Using GIS geoprocessing, these specific land classes were then extracted from the GAP/LANDFIRE dataset as shapefiles and utilized for further processing and analysis in the project map.

To better understand wetland classification and distribution across the landscape, another key habitat characteristic of the salamander species, spatial datasets were identified and downloaded from the FWS National Wetlands Inventory to identify the type, location, and dispersal of these habitat features. This spatial dataset includes wetland locations, classifications, size, and distributions for the conterminous United States (US Fish and Wildlife Service [US FWS], 2022). Similar to previous spatial datasets characterizing habitat parameters, data were downloaded and imported into GIS as shapefiles, which allowed for further extraction and geoprocessing within the project map. The FWS National Wetland Inventory is updated twice yearly; data utilized in this project were accessed during May 2023.

Using parameters of preferred habitat and distribution for the species, as identified by FWS species experts, spatial data were extracted, processed, and combined to create suitable land cover and wetland habitat layers. Since both the flatwoods salamander species utilize the same type of habitat, a single refined habitat map was created using the habitat features suitable for both species.

From the GAP/LANDFIRE National Terrestrial Ecosystem dataset, 25 ecological system land classifications that were identified through NatureServe ecological modeling as suitable habitat for the salamander species in Georgia were extracted. These 25 classes were extracted from the original dataset and converted into a new subset polygon, maintaining the metadata and attributes of the original file. These land classifications and their associated details are included in Table 2. Within the new polygon file, the ecological classes were merged to create a cohesive land classification layer and then clipped to the boundaries of the 11 Georgia counties of interest.

**TABLE 2:** Ecological land classifications and associated ID codes from the GAP/LANDFIRE National Terrestrial Ecosystems 2011 dataset utilized to define suitable land cover in the creation of refined habitat maps for reticulated and frosted flatwoods salamander species in the 11 Georgia counties restricted from Enlist Duo applications during 2022.

Ecological system class	ID code
Atlantic Coastal Plain Upland Longleaf Pine Woodland	3
East Gulf Coastal Plain Interior Upland Longleaf Pine Woodland—Open Understory Modifier	5
East Gulf Coastal Plain Interior Upland Longleaf Pine Woodland—Scrub/Shrub Modifier	6
East Gulf Coastal Plain Interior Upland Longleaf Pine Woodland—Offsite Hardwood Modifier	16
East Gulf Coastal Plain Near‐Coast Pine Flatwoods—Offsite Hardwood Modifier	17
Atlantic Coastal Plain Dry and Dry‐Mesic Oak Forest	33
Southern Atlantic Coastal Plain Mesic Hardwood Forest	39
Atlantic Coastal Plain Blackwater Stream Floodplain Forest—Forest Modifier	47
Atlantic Coastal Plain Small Blackwater River Floodplain Forest	49
Southern Coastal Plain Blackwater River Floodplain Forest	55
Southern Coastal Plain Seepage Swamp and Baygall	60
Atlantic Coastal Plain Nonriverine Swamp and Wet Hardwood Forest—Taxodium/Nyssa Modifier	61
Atlantic Coastal Plain Nonriverine Swamp and Wet Hardwood Forest—Oak Dominated Modifier	62
Southern Coastal Plain Nonriverine Basin Swamp	63
Southern Coastal Plain Nonriverine Basin Swamp—Okefenokee Bay/Gum Modifier	64
Southern Coastal Plain Nonriverine Basin Swamp—Okefenokee Pine Modifier	65
Southern Coastal Plain Nonriverine Basin Swamp—Okefenokee Taxodium Modifier	66
Atlantic Coastal Plain Southern Wet Pine Savanna and Flatwoods	69
East Gulf Coastal Plain Near‐Coast Pine Flatwoods—Open Understory Modifier	70
East Gulf Coastal Plain Near‐Coast Pine Flatwoods—Scrub/Shrub Understory Modifier	71
Southern Coastal Plain Nonriverine Cypress Dome	72
Atlantic Coastal Plain Depression Pondshore	85
East Gulf Coastal Plain Depression Pondshore	87
Southern Coastal Plain Nonriverine Basin Swamp—Okefenokee Clethra Modifier	90
Southern Coastal Plain Nonriverine Basin Swamp—Okefenokee Nupea Modifier	91

Abbreviation: ID, identification.

Following a similar procedure as land classification, the US FWS National Wetlands Inventory dataset was imported into the GIS software, and the appropriate wetland type of “Freshwater forested/shrub wetland” was selected, following consultation with FWS species experts. These selected features were exported to a new feature class and clipped to the boundaries of the restricted counties. Due to the preference of the species, any wetland feature that is not within 0.5 km of another wetland feature is considered unsuitable habitat (US Fish and Wildlife Service [US FWS], 2020a, 2020b). Wetland features that were not within 0.5 km of similar features were identified and removed from the dataset before continuing with further data processing.

Once suitable land cover, wetland features, and wetland distribution were identified and processed, areas where all habitat features were observed and present represented a suitable and potential habitat site for the species. Utilizing intersection geoprocessing tools in GIS software, a new layer was created and clipped to the boundaries of each restricted county, creating a refined habitat map that identified where all suitable parameters occurred for the species.

### 2.4 New methodology for calculating intersection of pesticide use and species habitat (overlap)

To calculate the overlap between potential Enlist Duo pesticide use on corn, cotton, and soybean and its interaction with flatwoods salamander habitat in Georgia, intersection analysis within GIS was performed between the processed cropland and habitat layers to identify and calculate where these parameters overlapped or touched. This then signaled a site where the species may be exposed to the pesticide application. Geometry calculation tools allowed for determining the sum of overlapping hectares of cropland with this habitat within each county. Identifying these sites enabled calculations of potential pesticide use site proximity to the habitat, allowing for a more precise understanding of the potential overlap occurring between the pesticide and the salamanders or their habitat.

## 3 RESULTS AND DISCUSSION

Considering the potential scale and widespread distribution of impacted agronomic cropland in Georgia from Enlist Duo use restrictions, created maps allowed for identification of the location and associated hectares of corn, cotton, and soybean that could no longer utilize the herbicide. Accessing the USDA CDL for a combined 5‐year period ensured that total hectare calculations reflected land use changes over time, specifically capturing crop rotations. When combined over 5 years, there were an estimated 366,353 cumulative ha (905,278 acres) of corn, cotton, and soybean within the 11 impacted counties. With over 366,000 ha (905,000 acres) of agronomically productive cropland unable to utilize the herbicide, this not only challenges a grower's ability to protect their yields and remain sustainable but also introduces a problematic precedent for pesticide‐ESA evaluations moving forward, potentially disrupting US agriculture production on a tremendous scale. With this amount of cropland impacted and accounting for over $500 million in total Farm Gate Value (as the total summary values of crops included in the analysis) from a restriction on a single pesticide product in 11 counties from one state, investigations into refining evaluation measures on pesticide–ESA interactions are warranted and necessary to continue supporting both species protection and agriculture production.

**FIGURE 1:**
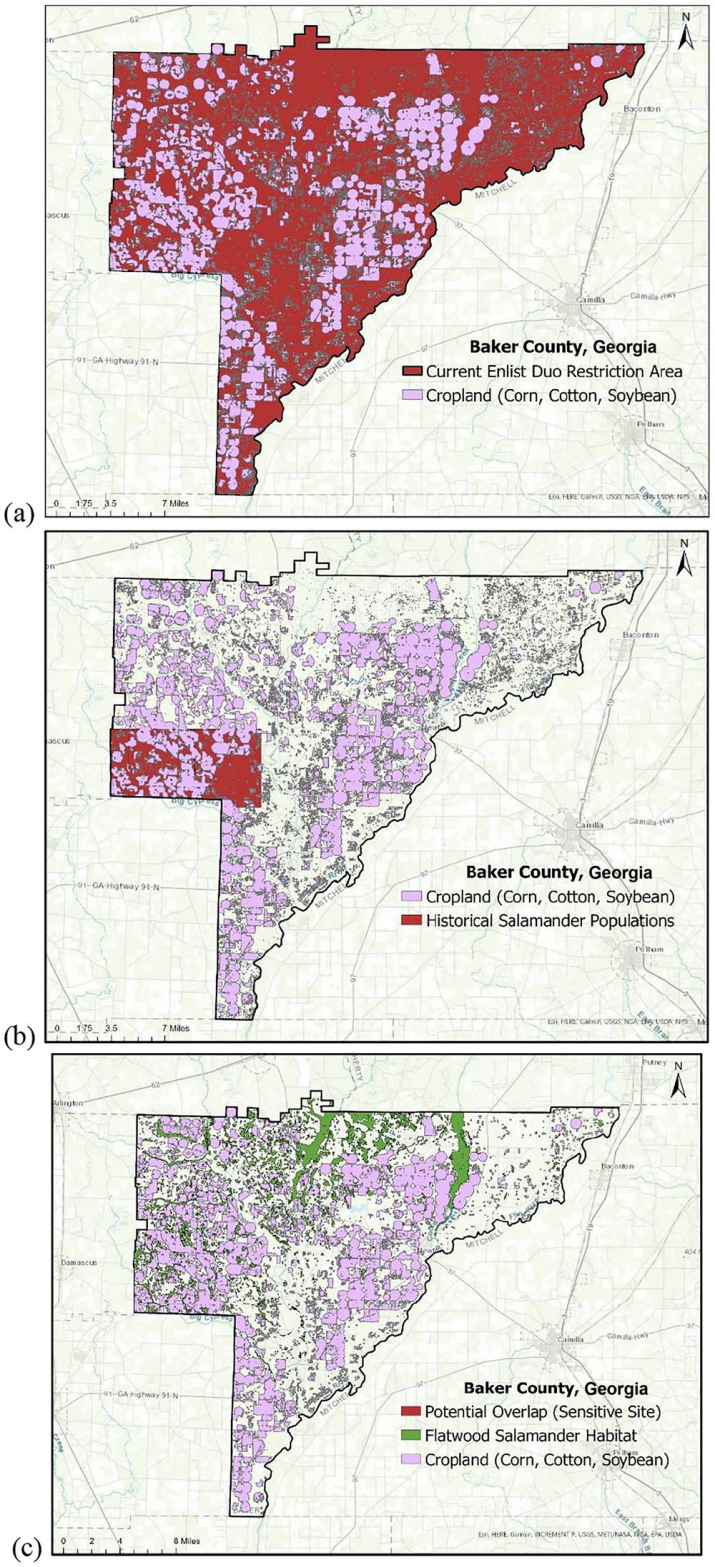
Map (a) highlights Enlist Duo restrictions (2022) in Baker County, GA, impacting 27,971 hectares of corn, cotton, and soybeans when combined over 2017 to 2021. Considering this cropland, 3,880 hectares or 13% of the identified cropland was located within the area identified as containing historical salamander populations (b). When Reticulated Flatwoods Salamander habitat was newly defined followed developed methodology, 32 hectares or 0.12% of cropland was identified as intersecting with area identified as a potential salamander habitat (c). Similar maps were created for each of the eleven restricted counties.

In determining where to begin in creating a refined map, basing restricted areas on sites with historical records of the flatwoods salamander species was deemed a logical starting place. While the exact recorded site of these species is protected, generalized locations of historically known populations, records, and observations are maintained and accessed through the Georgia Department of Natural Resources. Using the species’ generalized location information, along with the locations of corn, cotton, and soybean fields in each impacted county, maps were created that allowed for the spatial correlation between cropland and recorded historical species locations. Combined over 5 years, the total cropland (in hectares) found within each impacted county, the amount of area identified as historical locations of flatwoods salamander populations, and the calculated percent overlap between the two are provided in Table 3. For example, from 2017 to 2021, there were an estimated 27,971 ha (69,118 acres) of combined corn, cotton, and soybean hectares in Baker County, Georgia (Figure 1). Of this cropland, 3880 ha (9588 acres) were located within the area identified to have a historical population of reticulated flatwoods salamander species, indicating that 13% of restricted cropland overlapped with the historically recorded site of this species (Figure 1). Collectively, of the 366,353 ha (905,278 acres) prohibited from using Enlist Duo, the amount of actual farmland with historical salamander habitat equaled 27,992 ha (69,170 acres), or 8% of the total.

**TABLE 3:** Total corn, cotton, and soybean hectares falling within historical flatwoods salamander habitat, and the percent overlap of historical habitat with cropland for each of the 11 counties having Enlist Duo restrictions during 2022 in Georgia.

County^a^	Total cropland hectares^b^	Hectares within historical salamander habitat	Percent overlap (%)
Baker	27,971	3880	13.0
Berrien	29,213	2644	8.7
Brooks	34,667	1458	4.0
Burke	40,921	2150	4.9
Calhoun	22,196	0	0.0
Early	41,226	2096	4.8
Irwin	32,524	3137	9.4
Lee	25,070	10,251	38.0
Miller	36,113	1867	5.0
Screven	28,514	374	1.2
Worth	47,938	135	0.3
Total	366,353	27,992	7.64

^a^ Reticulated flatwoods salamanders historically recorded in Baker, Calhoun, Early, Lee, and Miller County, Georgia; frosted flatwoods salamander recorded in Berrien, Brooks, Burke, Irwin, Screven, and Worth County, Georgia.

^b^ Cropland includes corn, cotton, and soybean.

While basing a refined habitat map on the recorded historical presence of a species narrows the range and provides a more scientifically sound approach to identifying a species’ location, it does not account for unrecorded populations or other areas of suitable habitat that may be utilized by the species in the future. Regional FWS species experts shared insight into understanding how to protect a listed species in the long term begins with identifying and characterizing its habitat. Working closely with these experts, the habitat of the flatwoods salamander species was defined and characterized. Utilizing the previously defined datasets as advised by FWS cooperators, information on the specific land cover classifications (i.e., forest type/ecological), wetland classification, and distribution of wetlands that constitute a suitable habitat for the species were compiled and analyzed to determine where occupiable locations may exist across impacted counties and where these sites may also interact with potential pesticide use.

Once habitat was identified, this new location information was then overlaid with the locations of corn, cotton, and soybean fields to determine exactly where suitable flatwoods salamander habitat may intersect or border cropland where Enlist Duo could be applied. A refined map considering both cropland and potential habitat was created for each impacted county; the total cropland hectares in each county, hectares that overlap potential habitat, and the percent overlap of cropland and habitat are included in Table 4. For example, of the 27,971 ha (69,118 acres) of restricted cropland in Baker County, Georgia, 32 ha (79 acres) were located within or touching areas defined as habitat of the reticulated flatwoods salamander species (Figure 1). This indicates that 0.12% of productive cropland potentially overlaps with the habitat of this species within this county. Collectively, over the 11 impacted counties, the amount of combined farmland impacting potential species habitat equals 1219 ha (3012 acres), or 0.03% of total overlap.

**TABLE 4:** Total corn, cotton, and soybean hectares overlapping with newly defined potential habitat of the flatwoods salamander species, and the percent overlap of newly defined habitat with cropland for each of the 11 counties having Enlist Duo restrictions during 2022 in Georgia.

County^a^	Total cropland hectares^b^	Cropland overlapping with newly defined habitat	Percent overlap (%)
Baker	27,971	32	0.12
Berrien	29,213	108	0.37
Brooks	34,667	77	0.33
Burke	40,921	342	0.84
Calhoun	22,196	41	0.18
Early	41,226	96	0.23
Irwin	32,524	177	0.54
Lee	25,070	47	0.19
Miller	36,113	46	0.13
Screven	28,514	138	0.48
Worth	47,938	114	0.24
Total	366,353	1219	0.33

^a^ Reticulated flatwoods salamanders historically recorded in Baker, Calhoun, Early, Lee, and Miller County, Georgia; frosted flatwoods salamander recorded in Berrien, Brooks, Burke, Irwin, Screven, and Worth County, Georgia.

^b^ Cropland includes corn, cotton, and soybean.

The calculations, hectares, and maps presented in this study represent excerpts subject to the scale and resolution of the original data and should be interpreted as an example protocol of efficiently creating refined habitat maps to inform pesticide use limitation areas for future pesticide and Section 7 consultations under ESA compliance. While this work represents interactions between one pesticide, two endangered species, and 11 counties, there are many potential interactions that will come from the ESA‐pesticide consultation process once all pesticide products (1200+) and listed species (1600+) are evaluated. A problematic precedent was being set by placing proactive, broad county‐level restriction on pesticide use; however, since the completion of this work, the US EPA has moved to a more refined, species‐specific approach of defining pesticide use limitation areas, which incorporates suitable habitat, critical habitat, known locations, and buffers for potential negative effects from off‐target pesticide movement (particle drift and runoff/erosion) for all impacted species moving forward. Considering the tremendous implications ESA pesticides restrictions pose on the ability of farmers to produce a sustainable food supply, limiting pesticide use restrictions to exactly where listed species are located not only ensures the common goal of conserving species and habitat but also ensures that production agriculture can continue meeting the demand of a growing world population.

## AUTHOR CONTRIBUTIONS

**Taylor Randell‐Singleton**: Conceptualization; data curation; formal analysis; methodology; writing—original draft. **A. Stanley Culpepper**: Conceptualization; funding acquisition; resources; writing—review and editing.

## CONFLICT OF INTEREST STATEMENT

The authors declare no conflicts of interest.
